# The G-Protein-Coupled Estrogen Receptor (GPER/GPR30) in Ovarian Granulosa Cell Tumors

**DOI:** 10.3390/ijms150915161

**Published:** 2014-08-27

**Authors:** Sabine Heublein, Doris Mayr, Klaus Friese, Maria Cristina Jarrin-Franco, Miriam Lenhard, Artur Mayerhofer, Udo Jeschke

**Affiliations:** 1 Department of Obstetrics and Gynaecology, Ludwig-Maximilians-University of Munich, Campus Innenstadt, 80337 Munich, Germany; E-Mails: klaus.friese@med.uni-muenchen.de (K.F.); udo.jeschke@med.uni-muenchen.de (U.J.); 2 Department of Pathology, Ludwig-Maximilians-University of Munich, 80337 Munich, Germany; E-Mails: doris.mayr@med.uni-muenchen.de (D.M.); m.c.jarrinfranco@gmx.de (M.C.J.-F.); 3 Department of Obstetrics and Gynaecology, Ludwig-Maximilians-University of Munich, Campus Großhadern, 81377 Munich, Germany; E-Mail: miriam.lenhard@med.uni-muenchen.de; 4 Department of Anatomy III, Cell Biology, Ludwig-Maximilians-University of Munich, 80336 Munich, Germany; E-Mail: mayerhofer@lrz.uni-muenchen.de

**Keywords:** GPER, GPR30, ovarian granulosa cell tumor

## Abstract

Ovarian granulosa cell tumors (GCTs) are thought to arise from cells of the ovarian follicle and comprise a rare entity of ovarian masses. We recently identified the G-protein-coupled estrogen receptor (GPER/GPR30) to be present in granulosa cells, to be regulated by gonadotropins in epithelial ovarian cancer and to be differentially expressed throughout folliculogenesis. Thus, supposing a possible role of GPER in GCTs, this study aimed to analyze GPER in GCTs. GPER immunoreactivity in GCTs (*n* = 26; *n* (primary diagnosis) = 15, *n* (recurrence) = 11) was studied and correlated with the main clinicopathological variables. Positive GPER staining was identified in 53.8% (14/26) of GCTs and there was no significant relation of GPER with tumor size or lymph node status. Those cases presenting with strong GPER intensity at primary diagnosis showed a significant reduced overall survival (*p* = 0.002). Due to the fact that GPER is regulated by estrogens, as well as gonadotropins, GPER may also be affected by endocrine therapies applied to GCT patients. Moreover, with our data supposing GPER to be associated with GCT prognosis, GPER might be considered as a possible confounder when assessing the efficacy of hormone-based therapeutic approaches in GCTs.

## 1. Introduction

Ovarian granulosa cell tumors (GCTs) are classified as sex cord stromal tumors and comprise a rare entity of ovarian masses [1,2]. In spite of patients being mostly diagnosed at early stage disease and commonly presenting with a relatively favorable short-term prognosis after tumor resection [3], GCTs tend to relapse after decades. About 80% of patients with advanced stage disease die due to tumor recurrence [4]. Since the incidence of GCTs is low and as follow-up periods need to be extensive, randomized clinical trials on GCT treatment are missing [1]. Though, in the case of a non-resectable GCT, platinum-based chemotherapy is commonly applied; the therapeutic benefit of such an approach remains discussed controversially [5].

With GCTs deriving from steroid-producing granulosa cells, they may retain the ability to synthesize estrogens and inhibins. Further GCTs express steroid hormone, as well as gonadotropin receptors [6,7] and have been demonstrated to be responsive to pharmacological hormone ablation or receptor blockade in both animal models and humans [1,6,8]. Though endocrine therapies are well tolerated, a recent meta-analysis revealed response rates to be quite heterogeneous [1]. Van Meurs *et al*. [1] hypothesized that various response rates may be caused by different hormone receptor profiles of the respective primary tumor. To this direction, we recently highlighted the G-protein coupled estrogen receptor (GPER/GPR30) to be predictive for epithelial ovarian cancer (EOC) patient survival only in the case of missing co-expression of gonadotropin receptors [9]. We further provided *in vitro* evidence that gonadotropin receptor signaling may interfere with GPER action in epithelial ovarian cancer cells [9] and demonstrated GPER itself to be regulated by estrogen [10]. Hence, GPER, if present in GCTs, might be considered relevant as a possible confounder when assessing the efficacy of hormone-based therapeutic approaches in GCTs.

GPER, a G-protein-coupled estrogen receptor that has been demonstrated to mediate rapid estrogen signaling [11], has been shown to be of relevance in a range of cancer types deriving from reproductive [12,13,14,15], as well as non-reproductive [16,17] tissue. However, though GPER has been intensively studied in EOC, to the best of our knowledge, no report exists on GPER in ovarian granulosa cell tumors, so far. Therefore, this study aims to investigate whether GPER is present in GCTs and whether there is any correlation with clinicopathological parameters.

## 2. Results and Discussion

### 2.1. Patient Characteristics According to GPER Immunoreactivity

Twenty-six patients who had undergone surgery due to a GCT at the Department of Gynecology and Obstetrics at the Ludwig-Maximilians-University of Munich were included in the study. While 15 patients were identified with newly-diagnosed GCT, 11 patients underwent surgical resection of tumor relapse. In four cases, the tumor, as well as its recurrence was stained for GPER. Mean age at primary diagnosis was 54.8 ± 14.0 years.

The majority of the cases (65.2%; 15/23) were diagnosed with disease limited to the ovary (pT1), and there was no significant difference in tumor size or patient age when cases of primary diagnosed *vs*. recurrent tumors were compared. Lymph node involvement was only reported for nine cases with recurrent GCTs being found to be have spread to lymph nodes in five of five cases, whereas no lymph node positivity was reported for primary-diagnosed GCTs (*n* = 4) at all (*p* = 0.008).

**Figure 1:**
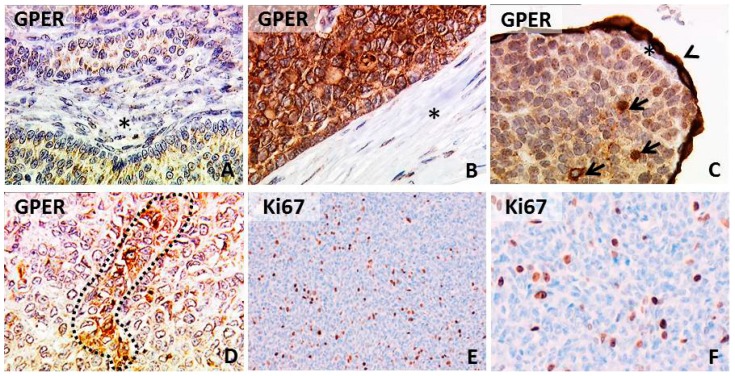
G-protein coupled estrogen receptor (GPER) and Ki67 immunoreactivity in GCTs. Micrographs of GPER in GCTs representing low (**A**) and high (**B**) uniform immunopositivity is shown; some cases presented high GPER staining intensity in single cells (marked by arrows (**C**)) or small foci (marked by a dotted line (**D**)). Ovarian stroma, as well as adjacent tumor stroma did not show GPER positivity (marked by stars). The ovarian surface epithelium presented strong GPER immunostaining (arrowhead (**C**)), as described before [18]. Representative Ki67 immunostaining is shown in (**E**,**F**). Magnification is: 100× in (**E**) and 250× in (**A**–**D**,**F**).

Positive GPER staining was observed in 53.8% (14/26) of cases with 26.9% (7/26) presenting with strong GPER intensity. GPER immunoreactivity ranged from uniform staining to single-cell positivity (Figure 1). Most positive cases showed either cytoplasmic or both membrane, as well as cytoplasmic staining (85.7%; 12/14). Immunoreactivity was not significantly altered when primary diagnosed *vs*. relapsed cases were compared. When this comparison was performed in a pairwise manner (*n* = 4), relapsed cases tended to show higher GPER immunoreactivity (*p* = 0.068). GPER was not associated with tumor size or patient age, neither in newly diagnosed nor in relapsed cases (Table 1). In addition, cases with strong GPER staining intensity were analyzed separately. However, no correlation to any of the clinicopathological parameters named above was noted (Table 2). Interestingly, a significant proportion of GCTs staining positive for GPER was found to co-express receptors for FSH and LH (Table 3).

GPER immunoreactivity was analyzed with respect to the mitotic status, as determined by Ki67 immunostaining (Table 3). In primary-diagnosed cases, strong GPER intensity (*int* = 3) showed a trend (*p* = 0.081) of being associated with a high mitotic index (Ki67 ≥ 10%). There was no association of GPER intensity and Ki67 in relapsed cases.

**Table 1:** Patient characteristics according to GPER immunoreactivity as analyzed by the IR-score. Patient characteristics subdivided by diagnosis and by GPER immunoreactivity as quantified by immunoreactive score (IRS) are displayed. Lymph node status (pN) was only available in few cases (*n* (initial diagnosis) = 4, *n* (recurrence) = 5), because no lymph node dissection was performed or because information was missing. Data were tested for independence by applying Fisher’s exact test, and *p*-values below 0.05 were considered statistically significant. IRS = immuno-reactive score ranging from zero (no immunoreactivity) to 12 (high immunoreactivity); na = not applicable; ns = not significant.

	Initial Diagnosis (*n* = 15)			Recurrence (*n* = 11)		
	GPER Negative (IRS ≤ 2; *n* = 7)	GPER Positive (IRS > 2; *n* = 8)	*p*	GPER Negative (IRS ≤ 2; *n* = 5)	GPER Positive (IRS > 2; *n* = 6)	*p*
pT						
pT1	5	6	ns	1	3	ns
pT2, pT3	2	1		4	1	
pN						
pN0	1	3	na	0	0	na
pN1	0	0		4	1	
subcellular localization						
cytoplasm	na	3	na	na	3	na
membr. + cytopl.	na	4		na	2	
nucleus	na	1		na	1	
distribution						
focal	na	7	na	na	4	na
uniform	na	1		na	2	
patient age						
≤54.8 years	3	2	ns	4	2	ns
>54.8 years	4	6		1	4	

**Table 2:** Patient characteristics according to GPER staining intensity. Patient characteristic subdivided by diagnosis and by GPER staining intensity. Lymph node status (pN) was only available in few cases (*n* (initial diagnosis) = 4, *n* (recurrence) = 5) since no lymph node dissection was performed or since information was missing. Data were tested for independence by applying Fisher’s exact test and *p*-values below 0.05 were considered statistically significant. *int* = staining intensity ranging from 0 (no staining) to 3 (strong intensity), na = not applicable, ns = not significant.

	Initial Diagnosis (*n* = 15)			Recurrence (*n* = 11)			
	GPER Negative (*int* ≤ 2; *n* = 12)	GPER Positive (*int* = 3; *n* = 3)	*p*	GPER Negative (*int* ≤ 2; *n* = 7)	GPER Positive (*int* = 3; *n* = 4)		*p*
pT							
pT1	10	1	ns	3	1		ns
pT2, pT3	2	1		4	1		
pN							
pN0	3	1	na	0	0		na
pN1	0	0		4	1		
subcellular localisation							
cytoplasm	na	1	na	na	1		na
membr. + cytopl.	na	2		na	2		
nucleus	na	0		na	1		
distribution							
focal	na	3	na	na	3		na
uniform	na	0		na	1		
patient age							
≤54.8 years	5	0	ns	4	2		ns
>54.8 years	7	3		3	2		

**Table 3:** *FSHR*, *LHCGR* and Ki67 in GPER-positive cases. Patients scored as GPER-positive (IRS > 2 or *int* = 3) were selected, and the expression of *FSHR* and *LHCGR* was determined by PCR in these samples. In addition, Ki67 immunoreactivity was analyzed.

	Initial Diagnosis		Recurrence	
	**GPER (*n* (IRS > 2) = 8)**	**GPER (*n* (*int* = 3) = 3)**	**GPER (*n* (IRS > 2) = 6)**	**GPER (*n* (*int* = 3) = 4)**
***FSHR***	5/8	2/3	4/6	3/4
***LHCGR***	6/8	2/3	4/6	3/4
**Ki67 (≥10%)**	3/8	2/3	2/6	1/4

### 2.2. Survival

**Figure 2:**
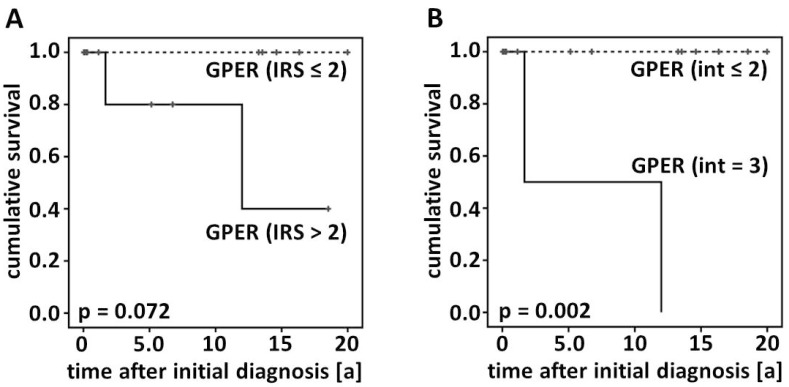
Overall survival according to GPER immunoreactivity. Kaplan–Meier graphs displaying the survival of GPER-positive *vs*. -negative cases by analyzing the IR-score (**A**) and by assessing GPER immunostaining intensity (**B**) are presented.

The impact on GPER on patients’ overall survival was analyzed in primary-diagnosed, as well as in relapsed GCTs. There was no difference among GPER-positive *vs*. -negative cases when Kaplan–Meier analysis was performed in those patients presenting with relapsed GCTs. However, the same analysis revealed GPER positivity (IRS > 2) tending to correlate with reduced overall survival in primary-diagnosed cases (*p* = 0.072). Those primary-diagnosed patients presenting strong GPER staining intensity (*int* = 3) showed a significantly reduced overall survival (*p* = 0.002) when compared with less intensely GPER expressing cases (Figure 2). Again, there was no significant relation in relapsed GCTs.

### 2.3. GPER and Hormone Receptor Insensitivity

The current study revealed GPER to be widely expressed in GCTs and demonstrated that high GPER intensity predicts poor outcome in newly-diagnosed GCT patients. With GPER being regulated by estrogens, as well as by gonadotropins, and since it may influence patients’ prognosis, it might be considered as a possible confounder when assessing the efficacy of hormone-based therapeutic approaches in GCT patients.

It could be argued that our conclusions are limited by the fact that the current study included just a small number of cases. Since GCTs account for about 5% of ovarian malignancies and are reported to have an incidence of 0.61 per 100,000 women per year, most studies report on just a few or even single patients [1]. However, a randomized multi-center study would be necessary to obtain an adequate sample size to prove whether our current findings are of clinical importance. Further, mutation analysis in the GCT samples studied may be an attractive way to clear whether GPER is associated with genetic aberrations observed in GCTs.

Besides a potential impact on patients’ prognosis predicted by GPER, the current study revealed that GPER immunoreactivity was not significantly altered, but even tended to be up-regulated when primary diagnosed and relapsed cases were compared, respectively. This phenomenon is of interest with respect to the fact that hormone receptor positivity may be lost during disease progression or chemotherapy [16,17]. Hormone receptor lost is regarded as a serious problem during the therapeutic management of cancer patients [17]. Since GPER expression remained unaltered, we hypothesize that it may continue to be targeted by anti-hormone-based therapies and, moreover, may be a stable, well-suited molecular target for novel therapeutic approaches. It needs to be noted that due to the rareness of GCTs, an optimal treatment strategy for advanced-staged GCTs has not been established, so far [1,4]. With patients being treated over decades and since these women might experience several relapses of their GCT, it is hard to retrospectively evaluate which chemo- or hormone therapies have been administered over time. However, we did not observe GPER loss in a randomly selected sample of GCTs and, thus, hypothesize that GPER expression is stable over time, even in the case that the patient received adjuvant chemotherapy.

### 2.4. Prognostic Significance of GPER in Ovarian Malignancies

The effect of GPER on cancer patients’ prognosis has been intensively studied during the last few years by several groups, achieving partly contradictory results. So far, there is no study dealing with the effect of GPER on GCT patients’ prognosis. Regarding EOC, Smith *et al*. published that patients highly expressing GPER are characterized by a significantly poorer outcome [13]. Later, it was speculated that GPER may not be related to EOC patients’ overall survival at all [19]. Only recently, several studies, including ours, revealed that GPER expression in EOC may be associated with a more favorable prognosis [9,20]. On the contrary, GPER was found to hold oncogenic activity in endometrial cancer [21].

The comparability of EOC and GCTs is rather limited, since EOC is regarded to derive from epithelial cells related to the ovarian surface or fallopian tube epithelium, while GCTs, stemming from granulosa cells, are ranged among female sex cord stromal tumors. Though no report exists on GPER in GCTs, GPER has already been studied in human seminoma. GPER is expressed in tumors of the testis [22,23], and new genetic polymorphisms have been identified in human seminoma [24]. In line with our observation of GPER being related to reduced patients’ overall survival in GCTs, GPER was demonstrated to induce proliferation in seminoma cells [12]. Larger studies and animal models are needed to confirm whether the relation of GPER to GCT patients’ prognosis reported herein might be of clinical relevance.

## 3. Experimental Section

### 3.1. Patients

Twenty-six patients that had undergone surgery due to a suspected ovarian tumor between 1987 and 2009 at the Department of Gynecology and Obstetrics at the Ludwig-Maximilians-University of Munich, Germany, were included in this study. Patients were treated according to the recommendations set by the German guidelines for the treatment of malignant ovarian tumors [25]. Patients underwent surgical resection of their GCT; especially in the case of a postmenopausal patient, primary surgery also included bilateral salpingo-oophorectomy, abdominal hysterectomy and omentectomy. In the case of incomplete tumor resection or of advanced-stage disease, most patients received an adjuvant platinum-based chemotherapy. Endocrine therapies were evaluated particularly for those patients presenting with low clinical performance. None of the patients had received neoadjuvant chemotherapy.

Following standard histological processing, tumor samples were classified as ovarian GCTs by qualified gynecological pathologist at our department. Histopathological diagnosis of all samples was confirmed by a second experienced gynecological pathologist (DM) before cases were finally included in the study. Patient’s clinical data were retrieved from patient charts, aftercare files and from the Munich Cancer Registry. The mean follow-up time of patients from primary diagnosis was 14.3 years (95% CI: 9.6–19.0), and the mean overall survival was 23.2 years (95% CI: 16.5–29.8). The outcome assessed was patient survival, with two deaths documented in the primary diagnosis group and six deaths registered in the recurrence group. Another three patients died due to reasons not related to their GCT and were thus handled as censored cases within survival analysis.

### 3.2. Ethical Considerations

The current study has been approved by the ethics committee of the Ludwig-Maximilians University Munich and has been carried out in compliance with the guidelines of the Helsinki Declaration of 1975. All specimens included in this study were left over samples collected during routine clinical diagnostics, and clinical diagnostics had already been fully completed when samples were retrieved for the study. Patient data were fully anonymized, and researchers were blinded for clinical information during experimental analysis.

### 3.3. Detection of GPER, Ki67, FSHR and LHCGR

GPER was detected by immunohistochemistry, as described before [9,10,18], and Ki67 immunohistochemistry was performed on a Ventana Benchmark XT autostainer, as explained elsewhere [26]. Normal ovarian tissue (GPER) and palatine tonsil tissue (Ki67) were used as positive controls. GPER immunoreactivity in GCTs was assessed by applying a semi-quantitative scoring system (immuno-reactive score (IRS)) by two independent observers by consensus. The IRS has been initially established for the assessment of estrogen and progesterone receptor positivity in breast cancer within routine histopathological diagnostics [27] and has been extensively used for assessing the immunoreactivity of numerous receptors, so far [18,28,29,30]. In brief, the IRS quantifies the intensity (1 = low, 2 = moderate, 3 = strong) and percentage of stained cells (0 =  no, 1 = less than 10%, 2 = 10%–50%, 3 = 51%–80%, 4 = 81%–100%). Multiplication of these sub-scores results in the IRS ranging from 0 to 12. In this study, GPER immunoreactivity, calculated as IRS > 2, was scored as positive, while those cases assigned an IRS ≤ 2 (negative or weak expression) were scored as negative. This cut off score has been extensively evaluated [28,29,31,32]. Further, samples presenting strong GPER staining intensity (*int* = 3) were analyzed separately. 

Ki67 immunostaining was quantified by assessing the percentage of positively-stained cells and samples were scored as positive in the case of ≥10% of stained cells.

*FSHR* and *LHCGR* expression in GPER positive GCTs was analyzed by RT-PCR. Following deparaffinization of GCT sections (2–3 µm) in a descending series of alcohols, mRNA was extracted by using the RNeasy FFPE Kit (Quiagen, Hilden, Germany), as per the manufacturer’s instructions, and total mRNA was subjected to reverse transcription employing SuperScript II reverse transcriptase (Invitrogen, Darmstadt, Germany). *FSHR* and *LHCGR* were amplified by using the following primers: *FSHR*-5' CTGCTCCTGGTCTCTTTGCT, 3' GGTCCCCAAATCCTGAAAAT; 5' nested GAGCTTGGGCTCAGGATGT, 3' nested GCACCTTTTTGGATGACTCG; *LHCGR*-5' TGGAGAAGATGCACAATGGA, 3' GGCAATTAGCCTCTGAATGG; 5' nested GCCTTCCGTGGGGCCACAG. Cycling conditions were: denaturation (94 °C for 60 s), annealing (54–62 °C for 30 s) and extension (at 72 °C for 60 s) for up to 35 cycles. PCR products were analyzed on a 2% agarose gel, and the identity of PCR products was verified by sequencing.

### 3.4. Statistical Analysis

Statistical analysis was performed by using SPSS v22.0 (IBM, Ehningen, Germany). The non-parametric Wilcoxon test was used for pairwise comparisons, while nominal data were tested for independence by Fisher’s exact test. The chi-square statistic of the log-rank test was calculated to test differences between survival curves of GPER-positive *vs.* -negative cases for significance. Data are presented as the mean ± standard deviation, and *p*-values below 0.05 were considered as statistically significant.

## 4. Conclusions

In conclusion, we demonstrate for the first time that GPER, being regulated by estrogens, as well as gonadotropins, is widely expressed in GCTs and that strong GPER staining intensity predicts a poor outcome in newly-diagnosed GCT patients. We thus hypothesize that GPER may also be affected by endocrine therapies applied to GCT patients. Therapies interfering with estrogen or gonadotropin activity are routinely applied to GCT patients. Hence, GPER might be considered as a possible confounder when assessing the efficacy of hormone-based therapeutic approaches in GCT cases.
